# Supplementary material for: Fast Phylogeny of SARS-CoV-2 by Compression
Source: Entropy (Basel). 2022 Mar 22;24(4):439. doi: 10.3390/e24040439 (PMC9030035; doi:10.3390/e24040439)
Supplement: Supplementary file 1 [file entropy-24-00439-s001.zip › entropy-1585159-SI.pdf]

Fast Whole-Genome Phylogeny by compression: the COVID-19 case

Rudi L. Cilibrasi and Paul M.B. Vitanyi

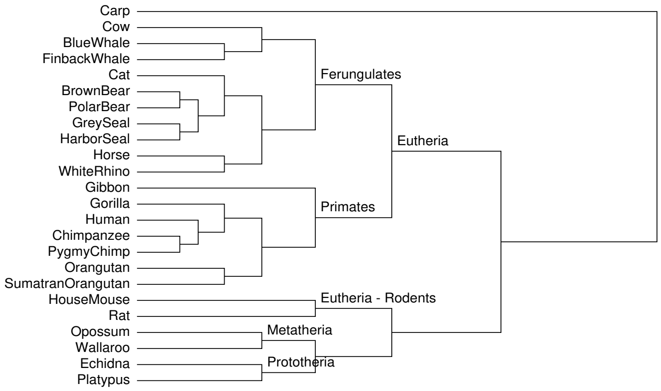

Figure S1. The evolutionary tree built from complete mammalian mtDNA sequences of 24 species, using the NCD matrix of Table SI. We have redrawn the tree from our output to agree better with the customary phylogeny tree format. The tree agrees exceptionally well with the NCD distance matrix: $S(T) = 0.996$.

I. Two Examples of the Compression Method

We treat two examples from [3] of the use of the Compression Method on known phylogenies.

The $S(T)$ value in the caption of Figures SI and S2 tells how well the tree represents the $n \times n$ NCD distance matrix of the n compared objects. To clarify, the n obects have $n \times n$ NCD distances. Hence they exist in n-dimensional space. Mapping that space onto two dimensions gives distortions of those distances, whatever way you do this. A flat map representing the earth sphere gives such problems. The Mercator projection is one way to do this with as consequence a particular distortion of the distances. In some areas this is a major distortion. A tree may represent the $n \times n$ distances between n objects easier than a more demanding 2-dimensional map. The $S(T)$ value tells how well these distances are preserved (0 is not at all and 1 is perfect). See how this is exactly calculated in reference [3].

A. *Mitochondrial DNA of 24 Species of Mammals*

We use the mitochondrial DNA of the following species: rat (*Rattus norvegicus*), house mouse (*Mus musculus*), grey seal (*Halichoerus grypus*), harbor seal (*Phoca vitulina*), cat (*Felis catus*), white rhino (*Ceratotherium simum*), horse (*Equus caballus*), finback whale (*Balaenoptera physalus*), blue whale (*Balaenoptera musculus*), cow (*Bos taurus*), gibbon (*Hylobates lar*), gorilla (*Gorilla gorilla*), human (*Homo sapiens*), chimpanzee (*Pan troglodytes*), pygmy chimpanzee (*Pan paniscus*), orangutan (*Pongo pygmaeus*), Sumatran orangutan (*Pongo pygmaeus abelii*), using opossum (*Didelphis virginiana*), wallaroo (*Macropus robustus*), platypus (*Ornithorhynchus anatinus*), Australian echidna (*Tachyglossus aculeatus*), brown bear (*Ursus arctos*), polar bear (*Ursus maritimus*), using the common carp (*Cyprinus carpio*) as the outgroup. We used the compressor PPMZ to obtain the NCD distance matrix in Table SI, and our quartet tree reconstruction method [4] to obtain the phylogeny tree Figure S2—that is, our own Complearn package [2].

Corresponding author: Paul M.B, Vitanyi; E-mail: paulv@cwi.nl

The mitochondrial genomes of the total of 24 species we used were downloaded from the GenBank Database on the world-wide web. Each is around 17,000 bp. The resulting phylogeny, with an almost maximal $S(T)$ score of 0.996 supports anew the currently accepted grouping (Rodents, (Primates, Ferungulates)) of the Eutherian orders [1] and additionally the Marsupionta hypothesis ((Prototheria, Metatheria), Eutheria), see Figure SI. Overall, our whole-mitochondrial NCD analysis supports the following hypothesis:

$$\overbrace{\underbrace{((primates, ferungulates)(rodents))}_{\text{Eutheria}}(Metatheria, Prototheria)}^{\text{Mammalia}},$$

which indicates that the rodents, and the branch leading to the Metatheria and Prototheria, split off early from the branch that led to the primates and ferungulates. Inspection of the distance matrix shows that the primates are very close together, as are the rodents, the Metatheria, and the Prototheria. These are tightly-knit groups with relatively close NCD's. The ferungulates are a much looser group with generally distant NCD's. The intergroup distances show that the Prototheria are furthest away from the other groups, followed by the Metatheria and the rodents. Also the fine-structure of the tree is consistent with biological wisdom.

B. *SARS Virus:*

We clustered the SARS virus after its sequenced genome was made publicly available, in relation to potential similar virii. The 15 virus genomes were downloaded from The Universal Virus Database of the International Committee on Taxonomy of Viruses, available on the world-wide web. The SARS virus was downloaded from Canada's Michael Smith Genome Sciences Centre which had the first public SARS Coronovirus draft whole genome assembly available for download (SARS TOR2 draft genome assembly 120403). The NCD distance matrix was computed using the compressor bzip2. The relations in Figure S2 are similar to the definitive tree based on medical-macrobio-genomics analysis, appearing later in the New England Journal of Medicine, [5]. We depicted the figure in the ternary tree style, rather than the genomics-dendrogram style, since the former is more precise for visual inspection of proximity relations.

References

[1] Y. Cao, A. Janke, P. J.Waddell, M.Westerman, O. Takenaka, S. Murata, N. Okada, S. Pbo, and M. Hasega, Conflict among individual mitochondrial proteins in resolving the phylogeny of Eutherian orders, *J. Mol. Evol.*, 47(1998), 307–322.

[2] R.L. Cilibrasi, The CompLearn Toolkit, 2003–, www.complearn.org

[3] R.L. Cilibrasi, P.M.B. Vitányi, Clustering by compression, *IEEE Trans. Information Theory*, 51:4(2005), 1523–1545.

[4] R. Cilibrasi, P.M.B. Vitanyi, A fast quartet tree heuristic for hierarchical clustering, *Pattern Recognition*, 44 (2011) 662–677.

	BlueWhale	BrownBear	Carp	Cat	Chimpanzee	Cow	Echidna	FinWhale	Gibbon	Gorilla	GreySeal	HarborSeal	Horse	HouseMouse	Human	Opossum	Orangutan	Platypus	PolarBear	PygmyChimp	Rat	SumOrang	Wallaroo	WhiteRhino
BlueWhale	0.005	0.906	0.943	0.897	0.925	0.883	0.936	0.616	0.928	0.931	0.901	0.898	0.896	0.926	0.920	0.936	0.928	0.929	0.907	0.930	0.927	0.929	0.925	0.902
BrownBear	0.906	0.002	0.943	0.887	0.935	0.906	0.944	0.915	0.939	0.940	0.875	0.872	0.910	0.934	0.930	0.936	0.938	0.937	0.269	0.940	0.935	0.936	0.923	0.915
Carp	0.943	0.943	0.006	0.946	0.954	0.947	0.955	0.952	0.951	0.957	0.949	0.950	0.952	0.956	0.946	0.956	0.953	0.954	0.945	0.960	0.950	0.953	0.942	0.960
Cat	0.897	0.887	0.946	0.003	0.926	0.897	0.942	0.905	0.928	0.931	0.870	0.872	0.885	0.919	0.922	0.933	0.932	0.931	0.885	0.929	0.920	0.934	0.919	0.897
Chimpanzee	0.925	0.935	0.954	0.926	0.006	0.926	0.948	0.926	0.849	0.731	0.925	0.922	0.921	0.943	0.667	0.943	0.841	0.946	0.931	0.441	0.933	0.835	0.934	0.930
Cow	0.883	0.906	0.947	0.897	0.926	0.006	0.936	0.885	0.931	0.927	0.890	0.888	0.893	0.925	0.920	0.931	0.930	0.929	0.905	0.931	0.921	0.930	0.923	0.899
Echidna	0.936	0.944	0.955	0.942	0.948	0.936	0.005	0.936	0.947	0.947	0.940	0.937	0.942	0.941	0.939	0.936	0.947	0.855	0.935	0.949	0.941	0.947	0.929	0.948
FinbackWhale	0.616	0.915	0.952	0.905	0.926	0.885	0.936	0.005	0.930	0.931	0.911	0.908	0.901	0.933	0.922	0.936	0.933	0.934	0.910	0.932	0.928	0.932	0.927	0.902
Gibbon	0.928	0.939	0.951	0.928	0.849	0.931	0.947	0.930	0.005	0.859	0.932	0.930	0.927	0.948	0.844	0.951	0.872	0.952	0.936	0.854	0.939	0.868	0.933	0.929
Gorilla	0.931	0.940	0.957	0.931	0.731	0.927	0.947	0.931	0.859	0.006	0.927	0.929	0.924	0.944	0.737	0.944	0.835	0.943	0.928	0.732	0.938	0.836	0.934	0.929
GreySeal	0.901	0.875	0.949	0.870	0.925	0.890	0.940	0.911	0.932	0.927	0.003	0.399	0.888	0.924	0.922	0.933	0.931	0.936	0.863	0.929	0.922	0.930	0.920	0.898
HarborSeal	0.898	0.872	0.950	0.872	0.922	0.888	0.937	0.908	0.930	0.929	0.399	0.004	0.888	0.922	0.922	0.933	0.932	0.937	0.860	0.930	0.922	0.928	0.919	0.900
Horse	0.896	0.910	0.952	0.885	0.921	0.893	0.942	0.901	0.927	0.924	0.888	0.888	0.003	0.928	0.913	0.937	0.923	0.936	0.903	0.923	0.912	0.924	0.924	0.848
HouseMouse	0.926	0.934	0.956	0.919	0.943	0.925	0.941	0.933	0.948	0.944	0.924	0.922	0.928	0.006	0.932	0.923	0.944	0.930	0.924	0.942	0.860	0.945	0.921	0.928
Human	0.920	0.930	0.946	0.922	0.667	0.920	0.939	0.922	0.844	0.737	0.922	0.922	0.913	0.932	0.005	0.949	0.834	0.949	0.931	0.681	0.938	0.826	0.934	0.929
Opossum	0.936	0.936	0.956	0.933	0.943	0.931	0.936	0.936	0.951	0.944	0.933	0.933	0.937	0.923	0.949	0.006	0.960	0.938	0.939	0.954	0.941	0.960	0.891	0.952
Orangutan	0.928	0.938	0.953	0.932	0.841	0.930	0.947	0.933	0.872	0.835	0.931	0.932	0.923	0.944	0.834	0.960	0.006	0.954	0.933	0.843	0.943	0.585	0.945	0.934
Platypus	0.929	0.937	0.954	0.931	0.946	0.929	0.855	0.934	0.952	0.943	0.936	0.937	0.936	0.930	0.949	0.938	0.954	0.003	0.932	0.948	0.937	0.949	0.920	0.948
PolarBear	0.907	0.269	0.945	0.885	0.931	0.905	0.935	0.910	0.936	0.928	0.863	0.860	0.903	0.924	0.931	0.939	0.933	0.932	0.002	0.942	0.940	0.936	0.927	0.917
PygmyChimp	0.930	0.940	0.960	0.929	0.441	0.931	0.949	0.932	0.854	0.732	0.929	0.930	0.923	0.942	0.681	0.954	0.843	0.948	0.942	0.007	0.935	0.838	0.931	0.929
Rat	0.927	0.935	0.950	0.920	0.933	0.921	0.941	0.928	0.939	0.938	0.922	0.922	0.912	0.860	0.938	0.941	0.943	0.937	0.940	0.935	0.006	0.939	0.922	0.922
SumOrangutan	0.929	0.936	0.953	0.934	0.835	0.930	0.947	0.932	0.868	0.836	0.930	0.928	0.924	0.945	0.826	0.960	0.585	0.949	0.936	0.838	0.939	0.007	0.942	0.937
Wallaroo	0.925	0.923	0.942	0.919	0.934	0.923	0.929	0.927	0.933	0.934	0.920	0.919	0.924	0.921	0.934	0.891	0.945	0.920	0.927	0.931	0.922	0.942	0.005	0.935
WhiteRhino	0.902	0.915	0.960	0.897	0.930	0.899	0.948	0.902	0.929	0.929	0.898	0.900	0.848	0.928	0.929	0.952	0.934	0.948	0.917	0.929	0.922	0.937	0.935	0.002

Table S1.

DISTANCE MATRIX OF PAIRWISE NCD. FOR DISPLAY PURPOSES, WE HAVE TRUNCATED THE ORIGINAL ENTRIES FROM 15 DECIMALS TO 3 DECIMALS PRECISION.

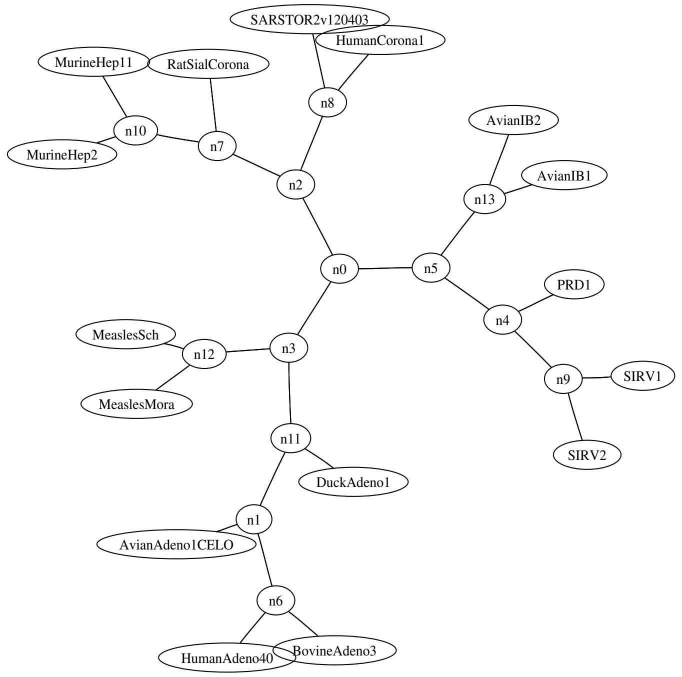

Figure S2. SARS virus among other viruses. Legend: AvianAdeno1CELO.inp: Fowl adenovirus 1; AvianIB1.inp: Avian infectious bronchitis virus (strain Beaudette US); AvianIB2.inp: Avian infectious bronchitis virus (strain Beaudette CK); BovineAdeno3.inp: Bovine adenovirus 3; DuckAdeno1.inp: Duck adenovirus 1; HumanAdeno40.inp: Human adenovirus type 40; HumanCorona1.inp: Human coronavirus 229E; MeaslesMora.inp: Measles virus strain Moraten; MeaslesSch.inp: Measles virus strain Schwarz; MurineHep11.inp: Murine hepatitis virus strain ML-11; MurineHep2.inp: Murine hepatitis virus strain 2; PRD1.inp: Enterobacteria phage PRD1; RatSialCorona.inp: Rat sialodacryoadenitis coronavirus; SARS.inp: SARS TOR2v120403; SIRV1.inp: Sulfolobus virus SIRV-1; SIRV2.inp: Sulfolobus virus SIRV-2. $S(T) = 0.988$.

[5] T.G. Ksiazek, et.al., A Novel Coronavirus Associated with Severe Acute Respiratory Syndrome, *New England J. Medicine*, Published at www.nejm.org April 10, 2003 (10.1056/NEJMoa030781).

II. DETAILS OF THE DATA-CLEANING PROCEDURE OF THE GISAID DATA IN THE MAIN TEXT

On the sequences initially downloaded from GISAID, we applied a lowercase transformation to each to reduce pointless variability. After that, we computed a histogram of all the characters in the sequence and counted the size of each group. Many sequences contained the base pairs A, C, G, N, T or other letters. We retained the viruses in the list after deduplication and filtering for A,C,G,T. This reduced the GISAID download to a set of unique 15,578 sequences with the known nucleotides A,C,G, and T. Each viral sequence is an RNA sequence and is around 30,000 RNA base pairs in size. The total size of all sequence data together is in the order of two gigabyte.

We then looked at whether there was much variation among the SARS-CoV-2 viruses themselves since this may invalidate the NCD distance between the inspected viruses and the selected SARS-CoV-2 virus. The worst NCD against the selected SARS-CoV-2 virus was 0.874027 namely gisaid hcov-19 2020 07 17 22.fasta¡hCoV-19/pangolin/Guangxi/P1E/2017 EPI ISL 410539/2017 from a Pangolin. Removing that one sequence from the list we got a worst NCD of 0.873367 also from a Pangolin in 2017.

Initially there are 15,430 sequences from GISAID that contain "hCov" in the name. We removed all sequences that contained /2017 in the name. After this we were left with 15,428 sequences and obtained a worst NCD of 0.738175 also from a Pangolin.

Removing the 21 sequences that contained /2017, /2018, or /2019 in the name, partially from Pangolins and possibly misclassified according to the 2020 criteria since the SARS-CoV-2 virus was only established in 2020, left 15,409 viruses in the list.

To clarify why there is the discrepancy below in that we obtain 15,578 imported sequences but when counting lose 100–200 in the next phase: There are some exact name duplicates in the GISAID data. When the "imported sequence" count is reported, then we count identically named identical sequences separately because they did both get imported (but one would overwrite the other). When counting the different names for sequences without /2017, /2018, /2019 in the name, these exact-name duplicates would collapse into a single one causing just one count.

III. NCD Distance Matrix underlying Figure 1 in the main body of the paper

DQ648856_Riboviria_3100 0.00344923 0.995585 0.789459 0.788631 0.700883 0.790701 0.787804 0.995632 0.793598 0.78739 0.932947 0.761714 0.795812 0.995632 0.941225 0.788769 0.784622 0.995344 0.788217 0.787666 0.78739 0.789321 0.795674 0.890453 0.796554 0.994546 0.933223 0.785084 0.793684 0.795674 0.788217 0.789321 0.791391 0.790011 0.812259 0.791898 0.76975 0.796278 0.804912 0.995447 0.789873 0.797022 0.793279 0.995477 0.931153 0.0862188 0.788907 0.787804 0.995344 0.796554 0.893902 0.790563 0.795755 0.997517 0.99531 0.788907 0.788493 0.791805 0.787666 0.78739 FJ938057_Coronaviridae_734 0.995585 0.00353107 0.995284 0.995305 0.996109 0.994992 0.995445 0.995632 0.995292 0.995306 0.99611 0.995452 0.995591 0.995495 0.995235 0.995028 0.995315 0.99561 0.995029 0.995166 0.995169 0.995159 0.995591 0.995843 0.995589 0.995975 0.995265 0.995313 0.995449 0.995591 0.995166 0.995146 0.995306 0.995167 0.995179 0.995258 0.99545 0.995589 0.995266 0.99548 0.995167 0.99545 0.995317 0.99561 0.99542 0.995586 0.995303 0.995306 0.99561 0.995451 0.996111 0.995306 0.995452 0.99774 0.995448 0.995146 0.995166 0.995155 0.995167 0.995169 EU371562_Riboviria_3206 0.789873 0.995839 0.00332871 0.0129816 0.769764 0.0134535 0.0375483 0.995768 0.0470849 0.0289895 0.921637 0.776185 0.788509 0.995768 0.937864 0.0277586 0.391897 0.995211 0.0280309 0.0301063 0.0284334 0.0283541 0.78782 0.880698 0.790076 0.994676 0.920111 0.391508 0.791063 0.78782 0.0256871 0.00582443 0.0695748 0.0345209 0.688981 0.407533 0.767958 0.790076 0.666158 0.9957 0.0401823 0.789989 0.789836 0.995211 0.917892 0.790868 0.0320486 0.0299558 0.995078 0.787043 0.877393 0.0503865 0.790794 0.997642 0.995448 0.00721221 0.0262395 0.0365449 0.0247169 0.0266391 EU371560_Riboviria_3204 0.789045 0.995857 0.0122911 0.00331446 0.770336 0.0187819 0.0314743 0.995768 0.0436404 0.0229155 0.921558 0.77591 0.787958 0.995768 0.93813 0.0214059 0.39176 0.995211 0.0218172 0.0238917 0.0222222 0.0233393 0.787269 0.880956 0.789524 0.994806 0.920177 0.391508 0.790787 0.787269 0.0197487 0.0111863 0.0647432 0.0283071 0.688567 0.407939 0.767682 0.789524 0.667449 0.995719 0.0339685 0.789437 0.789561 0.995211 0.917829 0.790178 0.0263776 0.0234677 0.995211 0.786768 0.878056 0.0443125 0.790243 0.997514 0.995448 0.00828615 0.0198868 0.0325922 0.018227 0.0202899 JX993987_Coronaviridae_778 0.698675 0.995832 0.767684 0.76854 0.00347367 0.768654 0.766703 0.995768 0.769561 0.767118 0.930119 0.705347 0.757371 0.995768 0.938586 0.76785 0.770566 0.995344 0.768158 0.766745 0.767288 0.76722 0.757647 0.874879 0.759201 0.994936 0.926497 0.772126 0.755896 0.755993 0.767435 0.767577 0.772639 0.769401 0.785813 0.778756 0.699435 0.759201 0.772544 0.995832 0.769815 0.758687 0.753891 0.995344 0.926162 0.701752 0.767371 0.768084 0.995211 0.757133 0.883734 0.770155 0.759509 0.997499 0.994759 0.767129 0.767574 0.768549 0.767191 0.76715 EU371564_Riboviria_3208 0.791115 0.995688 0.0137309 0.0196106 0.770599 0.00319933 0.0400331 0.995768 0.0501316 0.0316124 0.921923 0.777288 0.789474 0.995768 0.937822 0.0298301 0.393689 0.995078 0.0306545 0.0325922 0.0309179 0.0307054 0.788785 0.881391 0.791041 0.994546 0.920573 0.3933 0.791753 0.788785 0.0281729 0.0124809 0.0720596 0.036454 0.689669 0.409294 0.769199 0.791041 0.665322 0.995549 0.043082 0.790954 0.790525 0.995211 0.91839 0.792247 0.0346733 0.0325787 0.995078 0.788008 0.878039 0.0530094 0.791621 0.997496 0.99531 0.0140083 0.0290015 0.0393134 0.0270643 0.0292616 DQ640652_Riboviria_3098 0.788079 0.995721 0.0374103 0.0321645 0.768498 0.0398951 0.00331309 0.995632 0.0433462 0.0168415 0.92159 0.775358 0.786994 0.995632 0.937742 0.0215351 0.389142 0.995078 0.021397 0.0175318 0.0186335 0.0229155 0.786305 0.880453 0.78856 0.994676 0.920348 0.389302 0.78996 0.786443 0.0198785 0.03672 0.057979 0.0277471 0.687328 0.405636 0.767544 0.78856 0.66455 0.995721 0.0338211 0.78861 0.788734 0.995078 0.917863 0.789212 0.0173937 0.0229155 0.995078 0.786216 0.877554 0.0437604 0.78914 0.997515 0.99531 0.0369961 0.0200166 0.0320265 0.0200166 0.0194617 EF065506_Riboviria_3120 0.995359 0.995495 0.995359 0.995359 0.995768 0.995359 0.995495 0.0034125 0.995359 0.995359 0.995632 0.995905 0.995222 0.004914 0.995086 0.995086 0.995086 0.977917 0.995222 0.995222 0.995086 0.995222 0.995222 0.995768 0.995359 0.995195 0.994949 0.995086 0.995222 0.995222 0.995222 0.995222 0.995359 0.995359 0.995086 0.995123 0.995632 0.995222 0.995086 0.995086 0.995359 0.995222 0.995086 0.977917 0.994949 0.995359 0.995222 0.995222 0.977784 0.995086 0.996041 0.995359 0.995359 0.997679 0.97775 0.995359 0.995222 0.995359 0.995359 0.995222 FJ882954_Coronaviridae_725 0.794012 0.995707 0.0475003 0.0446071 0.771777 0.0504085 0.0436223 0.995768 0.00318515 0.0350635 0.923695 0.781836 0.792505 0.995632 0.940036 0.0335589 0.398236 0.995078 0.0280309 0.0343875 0.036853 0.0323651 0.791816 0.882149 0.794073 0.994546 0.92051 0.398263 0.795063 0.791816 0.0320398 0.046254 0.0771673 0.0403204 0.695317 0.413901 0.773473 0.794073 0.668467 0.995568 0.0252693 0.79385 0.79383 0.995211 0.919679 0.794592 0.0375743 0.0354776 0.995078 0.791316 0.878964 0.0339591 0.794653 0.997507 0.995172 0.0466694 0.032316 0.0185493 0.0321734 0.0321601 AY864805_Riboviria_3030 0.787528 0.995859 0.0282993 0.0230536 0.768636 0.0307841 0.0160133 0.995632 0.0342352 0.00317504 0.921452 0.774118 0.785754 0.995632 0.937604 0.0118719 0.383767 0.995211 0.0111817 0.00745445 0.00538302 0.0133904 0.785065 0.880177 0.787319 0.994806 0.920072 0.383926 0.788857 0.785203 0.0100773 0.0276091 0.0524572 0.0180839 0.68595 0.400488 0.766304 0.787319 0.66386 0.995721 0.0241579 0.787369 0.787633 0.995211 0.917587 0.788523 0.00869685 0.0125621 0.995078 0.7847 0.877278 0.0345113 0.788037 0.997653 0.995586 0.0278851 0.0100773 0.0223633 0.00980121 0.00910973 Beta-CoV_bat_Yunnan_RaTG13_2013—EPI_ISL_402131_EPI_ISL_402131 0.931843 0.995554 0.919834 0.920039 0.929008 0.920395 0.91952 0.995086 0.922448 0.919796 0.00361211 0.930402 0.927115 0.995086 0.95207 0.920453 0.917183 0.995078 0.919912 0.919348 0.920083 0.920609 0.92739 0.793543 0.927085 0.995066 0.443873 0.917149 0.926907 0.927253 0.919624 0.919567 0.921314 0.920188 0.918457 0.918846 0.929409 0.927223 0.924146 0.995276 0.920326 0.926917 0.926319 0.995078 0.441083 0.931853 0.919464 0.919796 0.995078 0.925706 0.793027 0.920486 0.926957 0.997638 0.995034 0.919556 0.919762 0.921096 0.919774 0.919393 DQ412043_Riboviria_3074 0.763782 0.995728 0.775358 0.775221 0.707001 0.776874 0.774531 0.995768 0.780871 0.773567 0.931229 0.00330761 0.747313 0.995768 0.938396 0.774945 0.768362 0.995211 0.775221 0.774118 0.773429 0.776461 0.746487 0.877343 0.748897 0.995066 0.92737 0.773291 0.741869 0.747589 0.774118 0.775634 0.779355 0.775083 0.789807 0.780111 0.0624311 0.748897 0.787073 0.995452 0.77715 0.748484 0.740256 0.995344 0.925579 0.727398 0.775358 0.77398 0.995211 0.747106 0.883958 0.778115 0.749173 0.997519 0.995039 0.774945 0.774118 0.778942 0.774118 0.773705 GQ153540_Coronaviridae_748 0.793883 0.995729 0.788785 0.788509 0.757647 0.7903 0.78782 0.995632 0.793194 0.786856 0.927115 0.746349 0.0033067 0.995495 0.938551 0.788647 0.782447 0.995211 0.787545 0.786856 0.787407 0.788647 0.0121246 0.807798 0.0166713 0.994546 0.926564

0.783274 0.291678 0.013089 0.78782 0.788647 0.79154 0.789198
0.799725 0.789459 0.73946 0.0158446 0.798154 0.995729 0.788923
0.0203913 0.289354 0.995211 0.924773 0.793331 0.788509
0.787545 0.995211 0.129788 0.835079 0.790025 0.0151557
0.997658 0.99504 0.788371 0.78782 0.790438 0.787269 0.787269
EF065507_Riboviria_3121 0.995359 0.995495 0.995359 0.995359
0.995768 0.995222 0.995495 0.0047775 0.995222 0.995222
0.995632 0.995768 0.995222 0.003276 0.995086 0.994949 0.995086
0.977784 0.995222 0.995222 0.995086 0.995222 0.995222 0.995768
0.995359 0.995066 0.994813 0.995086 0.995222 0.995222 0.995086
0.995086 0.995359 0.995222 0.995086 0.994987 0.995632 0.995222
0.995086 0.994949 0.995222 0.995222 0.995086 0.977784 0.994949
0.995359 0.995222 0.995222 0.977784 0.995086 0.995905 0.995222
0.995222 0.997543 0.97775 0.995359 0.995086 0.995222 0.995222
0.995222 NC_014470_Coronaviridae_823 0.940535 0.995656
0.935645 0.93592 0.939419 0.935596 0.935395 0.995632 0.938097
0.935533 0.952487 0.938534 0.939239 0.995632 0.00336323
0.936197 0.938404 0.994945 0.935791 0.935368 0.935404 0.936653
0.939102 0.946377 0.939628 0.994806 0.953621 0.938379 0.935733
0.939239 0.935506 0.935377 0.935809 0.935653 0.938567 0.939439
0.937543 0.939628 0.945419 0.995235 0.936067 0.939741 0.935408
0.994945 0.951978 0.940268 0.935626 0.935671 0.994945 0.93756
0.946381 0.936637 0.939636 0.997758 0.994897 0.935506 0.935506
0.937016 0.935653 0.935542 AY357076_Riboviria_2980 0.788769
0.995719 0.0265157 0.0209916 0.769507 0.0285872 0.0204307
0.995632 0.0325922 0.0110436 0.921972 0.775358 0.787269
0.995632 0.938268 0.00317636 0.386937 0.995211 0.009942
0.0117387 0.0129745 0.0117387 0.78658 0.881232 0.788835
0.994676 0.920729 0.387097 0.789684 0.786718 0.00690512
0.0251346 0.0559083 0.0168462 0.687603 0.403604 0.767407
0.788835 0.665516 0.995581 0.0227838 0.788886 0.788459
0.995211 0.918105 0.789764 0.0142246 0.0114578 0.995078
0.785941 0.878194 0.0332689 0.789416 0.997514 0.995448
0.0258252 0.00704323 0.0208535 0.00731842 0.00786749
KC881005_Coronaviridae_782 0.785035 0.995728 0.392173
0.392173 0.771255 0.394102 0.389831 0.995632 0.398787
0.384594 0.917872 0.768362 0.780794 0.995632 0.938955
0.388177 0.00316935 0.995078 0.386386 0.384594 0.385559
0.389417 0.781207 0.878876 0.782279 0.994416 0.919113 0.131735
0.781452 0.781207 0.386386 0.392449 0.396583 0.388866 0.67686
0.156076 0.760783 0.782417 0.655092 0.995728 0.39176 0.782141
0.779645 0.995211 0.916494 0.78848 0.388315 0.387075 0.995078
0.779523 0.881218 0.396031 0.783106 0.997382 0.995177 0.391897
0.386523 0.392724 0.385559 0.385697 EF065510_Riboviria_3124
0.995876 0.996408 0.99561 0.99561 0.996275 0.995477 0.99561
0.979247 0.995477 0.99561 0.996541 0.996009 0.995876 0.979247
0.995743 0.995344 0.995876 0.00305973 0.995477 0.995477
0.995477 0.995477 0.995876 0.996408 0.996009 0.995715
0.995876 0.995743 0.995743 0.995876 0.995477 0.995344 0.99561
0.995477 0.995344 0.99561 0.996009 0.996009 0.99561 0.996408
0.995477 0.995876 0.99561 0.00744978 0.995876 0.995876
0.995477 0.995477 0.00518824 0.99561 0.996275 0.995477
0.995876 0.997605 0.978981 0.99561 0.995477 0.995477 0.99561
0.995477 AY278741_Riboviria_2954 0.788355 0.995719 0.0274786
0.0220933 0.769539 0.0302403 0.0211209 0.995768 0.0270643
0.0111817 0.921569 0.775772 0.78658 0.995632 0.937724
0.0107705 0.385834 0.995078 0.003314 0.0110467 0.0131125
0.0117371 0.785891 0.88042 0.788146 0.994676 0.919635 0.38627
0.789822 0.785891 0.00883734 0.0263739 0.0560464 0.0167081
0.686639 0.402655 0.76782 0.788146 0.664181 0.995581 0.0169843
0.788196 0.788459 0.995211 0.917426 0.78935 0.014913 0.0115958
0.995078 0.785527 0.877244 0.0276091 0.788727 0.997514
0.99531 0.0269263 0.00897542 0.0150511 0.00842309 0.00814355
AY278554_Riboviria_2953 0.787804 0.995857 0.0295539
0.0241679 0.768264 0.0317636 0.0171176 0.995632 0.033697
0.00759249 0.921005 0.774807 0.785891 0.995632 0.93744
0.0124292 0.383767 0.995211 0.0110467 0.00317636 0.00952381
0.0140865 0.785203 0.880127 0.787319 0.994676 0.919624
0.383926 0.788857 0.785203 0.0106339 0.0287253 0.0521811
0.0191936 0.685813 0.400352 0.766993 0.787595 0.663444
0.995719 0.0240265 0.787507 0.787633 0.995211 0.917139 0.78935
0.010772 0.0139426 0.995078 0.784976 0.877227 0.0345113
0.788175 0.997514 0.995448 0.0290015 0.010772 0.0220964
0.0109086 0.01049 AY864806_Riboviria_3031 0.787666 0.995859
0.0277433 0.0223602 0.768668 0.0298137 0.0179434 0.995632
0.0360248 0.00510697 0.921739 0.774118 0.786167 0.995632
0.937474 0.0138026 0.38487 0.995078 0.0129745 0.00938578
0.0031746 0.0153209 0.785616 0.880607 0.78787 0.994546
0.920497 0.384753 0.789133 0.785616 0.0118703 0.0269151
0.0539683 0.0198758 0.686639 0.401165 0.766441 0.787733
0.664596 0.995721 0.0258109 0.787921 0.787908 0.995078
0.918012 0.788661 0.01049 0.0144928 0.995078 0.785389 0.877709
0.0360248 0.788451 0.997516 0.995448 0.0271912 0.0120083
0.0242926 0.0115942 0.010766 FJ882963_Coronaviridae_726
0.789321 0.995712 0.0278008 0.0238917 0.768603 0.0298755
0.0227775 0.995768 0.0318119 0.0136665 0.921992 0.777012
0.78782 0.995768 0.938589 0.0125673 0.388728 0.995211
0.0122894 0.0142246 0.015597 0.00318119 0.787131 0.880636
0.789524 0.994676 0.919502 0.388751 0.79065 0.787131
0.010772 0.0266943 0.0570127 0.0187793 0.68843 0.404146
0.768923 0.789524 0.663347 0.995712 0.0249931 0.789437
0.789423 0.995344 0.918396 0.79004 0.0161625 0.0142187
0.995211 0.78663 0.878008 0.0354776 0.789967 0.99751
0.995448 0.0272476 0.0109101 0.020332 0.0110467 0.01049
GQ153539_Coronaviridae_747 0.793607 0.995729 0.788371
0.788234 0.757647 0.789611 0.787407 0.995632 0.792643 0.786443
0.927528 0.745798 0.0122623 0.995632 0.938413 0.788234 0.78286
0.995211 0.787131 0.78658 0.786718 0.788234 0.0033067 0.807798
0.0238358 0.994676 0.926839 0.783825 0.291816 0.0121246
0.787545 0.788234 0.791127 0.788785 0.799725 0.78973 0.738771
0.0231469 0.79898 0.995729 0.788509 0.0271425 0.289492
0.995344 0.925048 0.792918 0.788096 0.787131 0.995344 0.131166
0.835079 0.789611 0.022458 0.997658 0.99504 0.787958 0.787545
0.790163 0.786856 0.786718 MG772933.1_bat_SL_CoVZC45
0.888245 0.995566 0.880837 0.881232 0.872662 0.88153 0.880039
0.995632 0.882011 0.880039 0.79285 0.876516 0.805732 0.995632
0.945684 0.881094 0.8786 0.995211 0.880282 0.879851 0.880469
0.880636 0.805594 0.00374117 0.806892 0.994676 0.785922
0.879101 0.801958 0.806283 0.880403 0.880698 0.882109 0.881248
0.889532 0.881994 0.875086 0.80703 0.888181 0.995427 0.88111
0.806536 0.801405 0.995344 0.783567 0.883846 0.880094
0.880177 0.995344 0.811854 0.306499 0.881419 0.806643
0.997506 0.994621 0.88056 0.880403 0.881506 0.880144 0.880193
GQ153545_Coronaviridae_753 0.794349 0.995727 0.790351
0.790076 0.759476 0.791868 0.789387 0.995632 0.794762 0.788284
0.92736 0.748071 0.0163957 0.995495 0.938939 0.790076 0.783933
0.995211 0.788973 0.788422 0.788835 0.790214 0.0234224
0.808959 0.00330806 0.994676 0.926533 0.784562 0.296347
0.0245247 0.789524 0.790076 0.793108 0.790627 0.80124
0.790814 0.741006 0.00399724 0.799035 0.995727 0.790489
0.0226051 0.294037 0.995344 0.924879 0.793522 0.789938
0.788973 0.995211 0.136733 0.835975 0.791454 0.0115766
0.997657 0.995038 0.7898 0.789249 0.792006 0.788835 0.788697
NC_025217_Coronaviridae_835 0.994546 0.996234 0.994546
0.994416 0.995325 0.994416 0.994546 0.995585 0.994416 0.994416
0.995585 0.995195 0.994936 0.995585 0.994936 0.994286 0.994157

0.995325 0.994286 0.994416 0.994286 0.994416 0.994936 0.995325
0.995066 0.00337618 0.994676 0.994286 0.994806 0.995066
0.994286 0.994286 0.994546 0.994416 0.994157 0.994157 0.994936
0.995066 0.994416 0.996364 0.994286 0.995066 0.994676 0.995325
0.994936 0.994546 0.994416 0.994286 0.995325 0.994936 0.995066
0.994286 0.995066 0.997792 0.995455 0.994546 0.994416 0.994286
0.994286 0.994416 selected_SARS_CoV_2_EPI_ISL_471246
0.932533 0.994986 0.918724 0.919072 0.925802 0.919182 0.918691
0.995086 0.91954 0.918829 0.444846 0.926681 0.926013 0.995086
0.952228 0.919486 0.91801 0.995078 0.918531 0.918381 0.919117
0.918257 0.92615 0.788416 0.925982 0.994546 0.00362117
0.917563 0.923045 0.92615 0.918658 0.918597 0.920486 0.919221
0.917493 0.91993 0.92541 0.925844 0.921053 0.994986 0.918945
0.925951 0.923151 0.995078 0.0111034 0.931577 0.918497
0.918553 0.995078 0.925706 0.791082 0.919244 0.92613
0.997632 0.994897 0.918447 0.918796 0.918605 0.918669
0.918565 KC881006_Coronaviridae_783 0.784946 0.995726
0.390405 0.390405 0.771712 0.392887 0.388337 0.995768 0.39716
0.38365 0.917838 0.772326 0.781758 0.995768 0.940033 0.387372
0.130219 0.995078 0.385856 0.383513 0.38434 0.3882 0.782171
0.879653 0.783184 0.994676 0.919355 0.00303281 0.782327
0.782171 0.385443 0.390543 0.396057 0.387924 0.67686 0.0382062
0.764819 0.783322 0.655087 0.995864 0.391508 0.783016 0.780609
0.995211 0.916736 0.789082 0.387372 0.386132 0.995211 0.781392
0.88172 0.395919 0.783765 0.997519 0.995451 0.39013 0.38558
0.391646 0.384615 0.384753 GQ153543_Coronaviridae_751
0.795339 0.995449 0.792442 0.792305 0.758516 0.793546 0.791753
0.995768 0.79658 0.79065 0.928148 0.740491 0.292643 0.995632
0.936009 0.791891 0.78283 0.995344 0.791477 0.79065 0.790925
0.792442 0.291816 0.804579 0.297037 0.994546 0.925252 0.784808
0.00330989 0.293194 0.791615 0.792305 0.794235 0.791339
0.800551 0.791492 0.73473 0.296623 0.798097 0.995449 0.792442
0.299641 0.0150117 0.995477 0.923459 0.79258 0.792305 0.791063
0.995344 0.292901 0.830782 0.793132 0.297409 0.997655
0.995035 0.791891 0.791477 0.794235 0.790787 0.790787
GQ153541_Coronaviridae_749 0.793745 0.995729 0.788647
0.788371 0.756407 0.789887 0.787683 0.995632 0.792643 0.786718
0.927528 0.746624 0.0132268 0.995632 0.938551 0.788509
0.782998 0.995211 0.787407 0.786718 0.787131 0.788509 0.011849
0.808212 0.0248002 0.994676 0.926702 0.7841 0.29278 0.0033067
0.78782 0.788509 0.791403 0.78906 0.799311 0.790001 0.73946
0.0241113 0.798567 0.995729 0.788785 0.0283825 0.290456
0.995211 0.925048 0.793056 0.788234 0.787407 0.995211 0.131992
0.834665 0.789749 0.0235602 0.997658 0.99504 0.788096 0.787683
0.7903 0.787131 0.786994 AY357075_Riboviria_2979 0.788217
0.995719 0.0245822 0.0196106 0.769093 0.0272062 0.0191883
0.995632 0.0312112 0.00980121 0.921143 0.774669 0.786856
0.995632 0.937578 0.00745753 0.385697 0.995078 0.00842309
0.0103577 0.0117322 0.0104958 0.786167 0.880541 0.788422
0.994676 0.919901 0.385856 0.789546 0.786305 0.00317636
0.0236155 0.0541137 0.0154653 0.686639 0.402249 0.766855
0.78856 0.663997 0.995581 0.0214029 0.788472 0.788321
0.995078 0.917415 0.78935 0.0128435 0.0102154 0.995078
0.785665 0.877503 0.0318885 0.78914 0.997514 0.995448
0.0238917 0.005386 0.0194724 0.00593759 0.00648723
EU371563_Riboviria_3207 0.789735 0.995701 0.00568576
0.0117387 0.769796 0.0122036 0.03672 0.995632 0.0458385
0.0280232 0.92137 0.776323 0.788234 0.995632 0.937595
0.0263776 0.392035 0.995078 0.0266501 0.0291396 0.0274672
0.026971 0.787545 0.88056 0.789938 0.994546 0.919845 0.391646
0.790925 0.787545 0.0241679 0.00318957 0.0688846 0.0330019
0.688705 0.407804 0.768234 0.7898 0.666066 0.995562 0.0388014
0.789713 0.789561 0.995078 0.917626 0.790868 0.0313579
0.0284373 0.995078 0.787043 0.87741 0.0490061 0.790518
0.997504 0.99531 0.00610179 0.0248584 0.035299 0.023198
0.0252588 AY515512_Riboviria_2987 0.791943 0.995721
0.0697129 0.0651574 0.773192 0.0717835 0.057979 0.995632
0.076339 0.0530094 0.922281 0.779906 0.790989 0.995632
0.937604 0.0567366 0.395618 0.995211 0.0565986 0.0527333
0.0546584 0.0574268 0.790163 0.882385 0.792557 0.994676
0.921452 0.396333 0.792718 0.7903 0.054804 0.0688846
0.00331309 0.0623965 0.693939 0.412546 0.772232 0.792557
0.669796 0.995721 0.0681944 0.792609 0.791351 0.995211
0.919382 0.792937 0.0557703 0.0574268 0.995211 0.7898 0.879763
0.0759249 0.793275 0.997653 0.99531 0.0690226 0.0554942
0.0662617 0.0554942 0.0549344 AY394850_Riboviria_2981
0.790149 0.995857 0.0342447 0.0288594 0.770782 0.0359017
0.0278851 0.995768 0.0396299 0.018222 0.921845 0.775772
0.787958 0.995768 0.937862 0.0176747 0.38859 0.995211
0.0168462 0.0193317 0.0202899 0.0186413 0.787269 0.881248
0.789524 0.994676 0.920326 0.388889 0.789546 0.787269
0.0157415 0.03314 0.0618443 0.003314 0.687466 0.405094
0.768372 0.789524 0.666529 0.995857 0.0295498 0.789575
0.788321 0.995211 0.918117 0.790868 0.0216791 0.0175318
0.995211 0.786216 0.878349 0.0400331 0.790105 0.997653
0.995586 0.0336924 0.0162938 0.0283071 0.0156034 0.0151829
KF569996_Coronaviridae_785 0.810882 0.995592 0.686364
0.686364 0.786226 0.687466 0.685399 0.995359 0.693113
0.683884 0.919008 0.788981 0.800275 0.995359 0.940083
0.685813 0.673967 0.995078 0.684573 0.683884 0.684573
0.686226 0.8 0.890083 0.801377 0.994416 0.918595 0.676446
0.801102 0.799449 0.684435 0.686226 0.691873 0.685399
0.00316804 0.683647 0.78416 0.801515 0.584573 0.995592
0.688705 0.801102 0.79989 0.995211 0.916529 0.810331 0.685262
0.684435 0.995078 0.795868 0.893802 0.690496 0.801791 0.997521
0.994766 0.686088 0.684711 0.690083 0.683884 0.683884
KF367457_Coronaviridae_784 0.791492 0.995665 0.405636
0.406043 0.778214 0.408075 0.404146 0.995665 0.411868 0.399268
0.919523 0.77835 0.787698 0.995665 0.9412 0.402926 0.153502
0.995078 0.401436 0.399133 0.399946 0.402926 0.787969 0.882807
0.789188 0.994286 0.921691 0.0364449 0.788917 0.788105
0.40103 0.405907 0.411597 0.403333 0.683918 0.00298063
0.771169 0.789324 0.664138 0.9958 0.406855 0.788917 0.786885
0.995078 0.919523 0.795285 0.402655 0.401707 0.995078 0.787698
0.885381 0.410784 0.78973 0.997426 0.995394 0.405772 0.401165
0.406584 0.400217 0.399946 DQ648857_Riboviria_3101 0.771681
0.995588 0.767407 0.767269 0.701503 0.769061 0.766717 0.995768
0.772232 0.76589 0.929546 0.0621555 0.741113 0.995768 0.937681
0.767269 0.760645 0.995211 0.767407 0.766441 0.76589 0.76851
0.740011 0.875776 0.74266 0.994936 0.925962 0.765371 0.736661
0.741113 0.766441 0.767682 0.771681 0.767544 0.78416 0.772253
0.0031711 0.742522 0.781745 0.99545 0.769199 0.742314 0.734885
0.995344 0.923894 0.738315 0.767682 0.766166 0.995211 0.741144
0.882256 0.77044 0.743109 0.997518 0.994899 0.766993 0.766579
0.770578 0.766166 0.766028 GQ153544_Coronaviridae_752
0.794211 0.995727 0.790351 0.790076 0.759476 0.791868 0.789387
0.995632 0.794762 0.788422 0.92736 0.748071 0.0158446 0.995495
0.939076 0.790076 0.783933 0.995211 0.789111 0.788422 0.788835
0.790076 0.0228713 0.808959 0.00413508 0.994546 0.926533
0.784562 0.295934 0.0239735 0.789387 0.790076 0.793108
0.790627 0.801377 0.790814 0.741006 0.00330806 0.799035
0.995727 0.790351 0.0220538 0.293761 0.995344 0.924879
0.793522 0.789938 0.788973 0.995211 0.136182 0.835975 0.791454
0.0110254 0.997657 0.995038 0.7898 0.789387 0.792006 0.788835
0.788697 JX993988_Coronaviridae_779 0.805464 0.995544
0.665049 0.666344 0.774489 0.664627 0.66317 0.995768 0.666667

0.662479 0.924701 0.785557 0.799118 0.995768 0.945976 0.664273 0.654954 0.994945 0.6628 0.662201 0.663216 0.662102 0.799532 0.889566 0.800138 0.994676 0.922027 0.655087 0.798373 0.799118 0.662616 0.664956 0.668415 0.665424 0.584573 0.664273 0.780367 0.800276 0.00334169 0.995823 0.665148 0.799779 0.797411 0.994945 0.921999 0.804801 0.663351 0.663584 0.994945 0.798897 0.893041 0.667725 0.800441 0.997494 0.995034 0.664771 0.66303 0.66376 0.66211 0.662526 AY646283_Riboviria_3003 0.995585 0.99548 0.995562 0.995581 0.995832 0.995549 0.995721 0.995086 0.995568 0.995583 0.996388 0.995865 0.995316 0.994949 0.995235 0.995581 0.995728 0.995876 0.995581 0.995581 0.995445 0.995712 0.995453 0.995982 0.995451 0.995975 0.995543 0.995726 0.995311 0.995453 0.995581 0.995424 0.995859 0.995581 0.995179 0.995529 0.995864 0.995451 0.995823 0.00330927 0.995581 0.99545 0.99518 0.995876 0.995697 0.995448 0.995718 0.995583 0.995876 0.995314 0.995833 0.995583 0.995452 0.997593 0.995448 0.995562 0.995581 0.99557 0.995581 0.995583 FJ882945_Coronaviridae_724 0.790011 0.995719 0.0401823 0.0346589 0.771334 0.0429439 0.0339591 0.995768 0.0249931 0.0245721 0.921845 0.777563 0.788096 0.995632 0.938277 0.0237503 0.391484 0.995078 0.0172604 0.0243027 0.026225 0.024855 0.787407 0.881386 0.789662 0.994676 0.919635 0.392335 0.790787 0.787407 0.0220933 0.0390776 0.0681944 0.0296879 0.690771 0.408481 0.769613 0.789662 0.666943 0.995581 0.003314 0.789713 0.789561 0.995078 0.917426 0.790592 0.0277548 0.0251242 0.995078 0.787181 0.877934 0.01836 0.790243 0.997514 0.99531 0.0396299 0.0222314 0.0129798 0.0218172 0.0218081 GQ153548_Coronaviridae_756 0.795505 0.995587 0.790954 0.790816 0.759239 0.792471 0.789989 0.995632 0.795229 0.788886 0.927192 0.747933 0.0210802 0.995632 0.939327 0.790816 0.784071 0.995211 0.789713 0.789024 0.789437 0.790816 0.0276936 0.80888 0.0234321 0.994676 0.926503 0.784946 0.299366 0.028658 0.789989 0.790678 0.793712 0.79123 0.800413 0.791085 0.740797 0.0228808 0.798952 0.995725 0.790954 0.00330943 0.29748 0.995211 0.92471 0.795091 0.790541 0.789713 0.995211 0.142385 0.836459 0.792195 0.0227398 0.997656 0.994898 0.790541 0.789851 0.792609 0.789437 0.7893 GQ153542_Coronaviridae_750 0.794794 0.995455 0.7908 0.790525 0.756645 0.792315 0.790112 0.995768 0.794794 0.78901 0.927834 0.739017 0.290456 0.995632 0.935822 0.790387 0.780884 0.995211 0.789974 0.789285 0.789285 0.7908 0.289905 0.804297 0.295276 0.994416 0.925217 0.783088 0.0152872 0.29142 0.789974 0.790662 0.792728 0.789836 0.799339 0.789866 0.733232 0.294725 0.797273 0.995455 0.790938 0.298031 0.00316761 0.995344 0.923289 0.791902 0.790662 0.789561 0.995344 0.291144 0.831428 0.791626 0.295689 0.997659 0.995042 0.790249 0.789836 0.792728 0.789285 0.789148 EF065511_Riboviria_3125 0.996009 0.996408 0.99561 0.99561 0.996275 0.995477 0.99561 0.97938 0.995477 0.99561 0.996541 0.996009 0.995876 0.979247 0.995743 0.995344 0.995876 0.00744978 0.995477 0.995477 0.995477 0.99561 0.995876 0.996541 0.996009 0.995715 0.995743 0.995743 0.995876 0.995876 0.995477 0.995477 0.995743 0.995477 0.995211 0.995743 0.996142 0.996009 0.995743 0.996408 0.995477 0.995876 0.995743 0.00319276 0.995876 0.995876 0.99561 0.995477 0.00558734 0.995743 0.996408 0.995477 0.996009 0.997738 0.978981 0.99561 0.995477 0.995477 0.995477 0.995477 MN908947.3_alt._SARS_CoV_2 0.93074 0.99542 0.916921 0.917139 0.925885 0.917418 0.916621 0.995086 0.919125 0.916759 0.442193 0.925028 0.924359 0.995086 0.951006 0.917415 0.915805 0.995211 0.916736 0.91631 0.917184 0.917704 0.924497 0.786061 0.924328 0.994806 0.011381 0.915357 0.921528 0.924635 0.916724 0.916794 0.918829 0.917288 0.915702 0.918304 0.923894 0.924466 0.921721 0.995281 0.91715 0.924297 0.921636 0.995211 0.00388619 0.930059 0.916563 0.916483 0.995211 0.924052 0.789591 0.917449 0.924614 0.997779 0.995172 0.916644 0.916724 0.918051 0.916874 0.916632 DQ412042_Riboviria_3073 0.0858049 0.995724 0.79004 0.789212 0.703407 0.791282 0.788385 0.995495 0.793765 0.787971 0.932956 0.725331 0.794985 0.995495 0.940957 0.789488 0.787378 0.995344 0.788936 0.788798 0.788109 0.789764 0.794158 0.885226 0.795038 0.994676 0.932267 0.788668 0.78996 0.794434 0.788936 0.789902 0.792247 0.790316 0.812121 0.794879 0.736936 0.795038 0.803835 0.995448 0.789902 0.796194 0.789561 0.995344 0.930197 0.00317285 0.789488 0.788247 0.995344 0.795038 0.891157 0.790316 0.794515 0.997517 0.995172 0.789488 0.789212 0.791971 0.788247 0.788109 AY278488_Riboviria_2951 0.788769 0.995718 0.0312198 0.0262395 0.768891 0.0337063 0.0165654 0.995632 0.0364691 0.00855881 0.920984 0.775772 0.786994 0.995632 0.93756 0.0146389 0.386937 0.995078 0.0144988 0.0103577 0.01049 0.0158862 0.786443 0.880232 0.788697 0.994676 0.919602 0.387235 0.790236 0.786305 0.0128435 0.0306672 0.054942 0.0212648 0.687603 0.403197 0.767958 0.78856 0.664595 0.995718 0.0272024 0.78861 0.78901 0.995211 0.917116 0.79004 0.00317723 0.0162893 0.995078 0.786216 0.877331 0.0374103 0.789278 0.997652 0.995448 0.0308054 0.0129816 0.0248653 0.0129798 0.0128364 AY278491_Riboviria_2952 0.788079 0.995859 0.0294036 0.0237438 0.769464 0.0321645 0.0222253 0.995632 0.0346494 0.0128382 0.9209 0.774394 0.786443 0.995632 0.937604 0.012286 0.386661 0.995211 0.0115958 0.0142187 0.0147688 0.0140806 0.785754 0.880453 0.788008 0.994676 0.919658 0.386959 0.789133 0.785754 0.0104914 0.0282993 0.0568747 0.0175318 0.686639 0.403197 0.766579 0.788008 0.664964 0.995583 0.0247101 0.787921 0.788046 0.995211 0.917311 0.788798 0.0168415 0.00317504 0.995078 0.784976 0.877554 0.0349255 0.788589 0.997653 0.995448 0.0287134 0.0107675 0.0230536 0.0102154 0.00979986 EF065512_Riboviria_3126 0.995876 0.996408 0.99561 0.99561 0.996142 0.995477 0.99561 0.979247 0.995344 0.995477 0.996408 0.996009 0.995876 0.979247 0.995743 0.995344 0.995876 0.00518824 0.995477 0.995344 0.995477 0.995477 0.995743 0.996408 0.996009 0.995715 0.995743 0.995743 0.995743 0.995876 0.995344 0.995344 0.99561 0.995477 0.995211 0.99561 0.996009 0.996009 0.99561 0.996408 0.995477 0.995876 0.99561 0.00558734 0.995743 0.995876 0.995477 0.995477 0.00305973 0.99561 0.996275 0.995477 0.995876 0.997605 0.978848 0.99561 0.995344 0.995477 0.995477 0.995477 GQ153547_Coronaviridae_755 0.794624 0.995589 0.787733 0.787319 0.757409 0.789111 0.786906 0.995495 0.791868 0.785665 0.926809 0.746692 0.130201 0.995495 0.938249 0.787181 0.781452 0.995211 0.786354 0.785665 0.786354 0.787319 0.131303 0.81337 0.137009 0.994676 0.927223 0.783184 0.294142 0.132268 0.786492 0.787457 0.790351 0.787457 0.795592 0.789188 0.740041 0.13632 0.798759 0.995589 0.788146 0.141833 0.291971 0.995211 0.925569 0.793384 0.787457 0.786216 0.995211 0.00317023 0.835837 0.788973 0.136852 0.997657 0.995038 0.787181 0.786354 0.789662 0.785941 0.785803 MG772934.1_bat_SL_CoVZXC21 0.892936 0.995972 0.876976 0.877503 0.882345 0.877622 0.876588 0.995632 0.877579 0.876449 0.793721 0.881753 0.833287 0.995632 0.94527 0.877503 0.880529 0.995344 0.876553 0.87626 0.877019 0.876902 0.83315 0.308716 0.834459 0.994676 0.792054 0.880755 0.829954 0.833012 0.876674 0.876855 0.878796 0.877382 0.893113 0.88362 0.879912 0.834459 0.890818 0.995555 0.87683 0.834528 0.830326 0.995477 0.790423 0.89033 0.876364 0.876726 0.995477 0.835286 0.00361161 0.877278 0.834206 0.997639 0.994759 0.876699 0.876813 0.877215 0.876415 0.876605 FJ882942_Coronaviridae_723 0.790701 0.995721 0.0505246 0.0451408 0.771811 0.0530094 0.0437604 0.995768 0.033545 0.0349255 0.922004 0.778666 0.789474 0.995632 0.938984

0.0342352 0.39548 0.995078 0.0281612 0.0349255 0.0365769 0.0353396 0.788647 0.881833 0.791041 0.994546 0.92021 0.396333 0.791615 0.788647 0.0327167 0.0494202 0.0757869 0.0401712 0.693113 0.412139 0.771129 0.791041 0.669658 0.995583 0.0184981 0.791092 0.790387 0.995078 0.918001 0.791006 0.0383766 0.0354776 0.995078 0.788284 0.878796 0.00317504 0.791621 0.997377 0.99531 0.0498343 0.0328548 0.024296 0.0325787 0.0318841 GQ153546_Coronaviridae_754 0.793688 0.995728 0.790932 0.790656 0.759647 0.792448 0.789967 0.995632 0.795342 0.788864 0.927233 0.748208 0.015569 0.995632 0.938809 0.790656 0.784622 0.995211 0.789691 0.789002 0.789416 0.790794 0.022458 0.808848 0.0118523 0.994676 0.926681 0.785143 0.296996 0.0235602 0.789967 0.790794 0.793688 0.791207 0.801515 0.791356 0.741455 0.011301 0.799201 0.995728 0.791069 0.0219129 0.294725 0.995344 0.925028 0.792861 0.790518 0.789553 0.995344 0.137128 0.835584 0.792172 0.00330761 0.997657 0.995039 0.79038 0.789967 0.792585 0.789416 0.789278 humanMito 0.998482 0.998729 0.998197 0.998205 0.998333 0.998053 0.998067 0.998771 0.998061 0.998205 0.999028 0.998484 0.998347 0.998635 0.998178 0.997928 0.998071 0.998138 0.998067 0.998067 0.998068 0.998064 0.998209 0.998753 0.998346 0.998182 0.998189 0.998208 0.998345 0.998209 0.997928 0.99792 0.998343 0.998067 0.997934 0.998103 0.998346 0.998346 0.997911 0.998646 0.998067 0.998345 0.99821 0.998271 0.998334 0.998345 0.998066 0.998067 0.998138 0.998346 0.99875 0.998067 0.998346 0.00602107 0.998069 0.998058 0.998067 0.998062 0.998067 0.998068 NC_034440_Coronaviridae_847 0.994897 0.995448 0.995448 0.995448 0.995172 0.995172 0.99531 0.978569 0.99531 0.99531 0.995724 0.99559 0.995178 0.978569 0.99531 0.995172 0.995177 0.977784 0.995172 0.995172 0.995172 0.99531 0.99504 0.995172 0.995176 0.995455 0.995034 0.995175 0.995035 0.995178 0.995034 0.995172 0.995172 0.99531 0.994766 0.995258 0.995312 0.995314 0.994897 0.995586 0.99531 0.995174 0.994904 0.977917 0.995172 0.994759 0.99531 0.99531 0.977917 0.995038 0.995172 0.995172 0.995176 0.997379 0.00331034 0.995448 0.995172 0.995172 0.995172 0.995172 EU371561_Riboviria_3205 0.789183 0.9957 0.0073509 0.00925287 0.769209 0.0137309 0.0368581 0.995768 0.046254 0.0282993 0.921082 0.775634 0.78782 0.995768 0.937725 0.0267919 0.391622 0.995211 0.0272024 0.0292777 0.0276052 0.0273859 0.787131 0.880421 0.789249 0.994676 0.919834 0.391232 0.790512 0.787131 0.0245822 0.00624047 0.0690226 0.0336924 0.68843 0.407668 0.767407 0.789387 0.665881 0.9957 0.0394919 0.7893 0.789285 0.995211 0.917337 0.790316 0.0314961 0.0289895 0.995078 0.786492 0.877254 0.0495583 0.789967 0.997503 0.995448 0.00332871 0.0254109 0.0358527 0.0237503 0.0256729 AY350750_Riboviria_2977 0.788493 0.995857 0.0254109 0.0198868 0.769093 0.0280348 0.0194644 0.995632 0.0316255 0.00993926 0.921282 0.774669 0.78658 0.995632 0.937578 0.00773374 0.385834 0.995211 0.00869925 0.0106339 0.0118703 0.0104958 0.785891 0.880679 0.788146 0.994676 0.920039 0.385994 0.78927 0.785891 0.005386 0.024306 0.0550801 0.0157415 0.686639 0.40252 0.766855 0.788284 0.664411 0.995719 0.0216791 0.788058 0.788046 0.995211 0.917553 0.789488 0.0129816 0.0103534 0.995211 0.785527 0.877641 0.0323026 0.788864 0.997514 0.995448 0.0247203 0.00317636 0.0197487 0.00607567 0.00676329 FJ882935_Coronaviridae_722 0.792219 0.995709 0.0366833 0.0335589 0.77021 0.039175 0.0321645 0.995768 0.0185493 0.0234677 0.922619 0.779768 0.789611 0.995768 0.939092 0.0222345 0.392449 0.995211 0.0160177 0.0227869 0.0252588 0.0208852 0.788923 0.881783 0.791178 0.994676 0.919712 0.392749 0.79258 0.788923 0.0204392 0.0355759 0.0663998 0.0288594 0.692287 0.408481 0.771543 0.791178 0.665144 0.99557 0.0133941 0.79123 0.791351 0.995211 0.918605 0.792937 0.0256942 0.0237438 0.995078 0.788835 0.878599 0.024434 0.791759 0.997508 0.995448 0.0359911 0.0207154 0.00332226 0.0208506 0.0205659 EU371559_Riboviria_3203 0.787804 0.995719 0.0240265 0.0185032 0.768848 0.0266501 0.0197405 0.995632 0.0316211 0.00980121 0.921292 0.774531 0.786167 0.995632 0.937862 0.008285 0.384732 0.995078 0.008285 0.0110467 0.0118703 0.0106324 0.785616 0.88042 0.78787 0.994546 0.919912 0.385167 0.788857 0.785616 0.00635184 0.0229218 0.0550801 0.0151892 0.685813 0.401707 0.766579 0.78787 0.663629 0.995719 0.021541 0.787921 0.787633 0.995211 0.917564 0.788661 0.0135322 0.0103534 0.995078 0.784976 0.877382 0.0320265 0.788589 0.997514 0.995448 0.0233361 0.006628 0.019884 0.003314 0.00676329 NC_004718_Coronaviridae_806 0.787528 0.995859 0.0258109 0.0204279 0.768806 0.0285714 0.0190476 0.995632 0.03147 0.0089717 0.921049 0.774118 0.786029 0.995632 0.937474 0.00869565 0.385008 0.995078 0.00814355 0.01049 0.0109041 0.0102139 0.78534 0.880331 0.787595 0.994676 0.919531 0.385167 0.788857 0.785478 0.00690131 0.0249827 0.0545204 0.0151829 0.68595 0.401436 0.766441 0.787733 0.664044 0.995721 0.021256 0.787645 0.787633 0.995078 0.917184 0.788523 0.0131125 0.00952381 0.995078 0.784838 0.877295 0.03147 0.788313 0.997516 0.995448 0.0252588 0.00703934 0.0197378 0.00676329 0.0031746

IV. NCD Distance Matrix underlying Figure 2 in the main body of the paper

EPI_ISL_402132_19_Wuhan_HBCDC-HB-01_2019 0.00375156 0.0115326 0.442623 0.0393219 0.00763253 0.00569049 0.43407 0.0106989 0.786061 0.0108378 0.00500208 0.736279 0.00430735 0.00693963 1.5134e-08 0.872725 0.87203 0.00624566 0.00652325 0.0113936 0.0111157 0.00569049 0.00639066 0.01056 0.00625174 0.00569049 0.00680745 0.00693963 0.789415 0.997638 0.0113936 0.0113936 0.00680839 0.0113936 0.0113936 0.0112547 0.00486246 EPI_ISL_421342_19_USA_WI-UW-83_2020 0.0111157 0.00376097 0.445402 0.0417885 0.0137386 0.0117974 0.435715 0.0044562 0.788139 0.00431755 0.0113936 0.738453 0.01056 0.0129077 2.14204e-08 0.873749 0.873089 0.0122137 0.0126301 0.00487465 0.00445745 0.0117974 0.0123645 0.00501253 0.0123645 0.0117974 0.0123645 0.0130465 0.790943 0.997632 0.00487465 0.00487465 0.0112656 0.00487465 0.00487465 0.00487465 0.0108363 Beta-CoV_bat_Yunnan_RaTG13_2013—EPI_ISL_402131_EPI_ISL_402131 0.441234 0.444429 0.00361211 0.461795 0.441715 0.441083 0.486385 0.443873 0.793543 0.443873 0.44165 0.737983 0.441234 0.441915 7.40867e-07 0.87955 0.878161 0.44136 0.441499 0.444012 0.444151 0.441083 0.441373 0.444012 0.441928 0.441083 0.441512 0.442193 0.793027 0.997638 0.444012 0.443457 0.442484 0.444429 0.444012 0.443457 0.441095 EPI_ISL_412900_19_Wuhan_HBCDC-HB-04_2019 0.0401556 0.0427636 0.463323 0.00383632 0.043436 0.041499 0.453936 0.0423339 0.797561 0.0422006 0.0393219 0.750556 0.0400167 0.0426093 7.24103e-08 0.878615 0.877954 0.041499 0.0406662 0.0422006 0.0422123 0.041499 0.0418172 0.0420496 0.0419561 0.041499 0.0414004 0.041499 0.801639 0.997442 0.0427577 0.0427577 0.0392211 0.0427577 0.0427577 0.0426184 0.0405668 EPI_ISL_402123_19_Wuhan_IPBCAMS-WH-01_2019 0.0077713 0.0141549 0.442687 0.0424646 0.00374688 0.00610602 0.435332 0.013461 0.786338 0.013461 0.00791007 0.735776 0.00707744 0.00721621 1.76951e-08 0.873161 0.872329 0.00666112 0.00679989 0.0138773 0.0137386 0.0062448 0.00832639 0.0131835 0.00832639 0.00610602 0.00846517 0.00735498 0.789759 0.997641 0.0141549

0.0141549 0.00957535 0.0141549 0.0141549 0.0138773 0.00693866 EPI_ISL_403929_19_Wuhan_IPBCAMS-WH-04_2019 0.00541291 0.0117974 0.442193 0.0403886 0.00596725 0.0037474 0.434559 0.0111034 0.785922 0.0112422 0.0055517 0.735878 0.00471895 0.00499653 1.42027e-08 0.873005 0.872311 0.00444136 0.00458015 0.0117974 0.0115198 0.00388619 0.00624566 0.0109646 0.00596808 0.0037474 0.00610687 0.00513532 0.789591 0.997779 0.0119362 0.0119362 0.00721721 0.0117974 0.0117974 0.0116586 0.00458015 EPI_ISL_412977_19_bat_Yunnan_RmYN02_2019 0.433097 0.435158 0.486663 0.452401 0.434638 0.433865 0.00348967 0.434619 0.793266 0.43468 0.433375 0.752226 0.433236 0.434698 7.27363e-07 0.882369 0.881151 0.434282 0.434004 0.434819 0.434801 0.433865 0.434148 0.434976 0.433732 0.433865 0.434287 0.435115 0.79136 0.997627 0.435097 0.434262 0.433658 0.435237 0.434958 0.434958 0.433315 EPI_ISL_461437_19_USA_UNKNOWN-UW-5620_2020 0.010421 0.0044562 0.444846 0.0413591 0.0129059 0.0109646 0.435037 0.00362067 0.788278 0.00375992 0.01056 0.738314 0.00972627 0.0120749 2.04891e-08 0.873749 0.873089 0.0115198 0.0116586 0.00431695 0.00403843 0.0109646 0.011531 0.00431635 0.011531 0.0109646 0.011531 0.0122137 0.791082 0.997633 0.00431695 0.0044562 0.0105702 0.00431695 0.00431695 0.00417769 0.0101417 MG772933.1_bat_SL_CoVZC45 0.783844 0.785645 0.79285 0.795206 0.783844 0.783567 0.793404 0.785784 0.00374117 0.785922 0.783844 0.831509 0.783705 0.782874 1.3134e-06 0.897049 0.898296 0.783705 0.783567 0.785922 0.786199 0.783567 0.783705 0.785645 0.784675 0.783567 0.784121 0.784121 0.306499 0.997506 0.785922 0.786338 0.784398 0.786061 0.785922 0.786061 0.783844 selected_SARS_CoV_2_EPI_ISL_471246 0.010421 0.00417827 0.444846 0.0412256 0.0130447 0.0111034 0.435097 0.00375992 0.788416 0.00362117 0.0106989 0.738314 0.00986522 0.0122137 2.04891e-08 0.873888 0.873228 0.0115198 0.0117974 0.004039 0.00389972 0.0111034 0.0116699 0.00417711 0.0116699 0.0111034 0.0116699 0.0123525 0.791082 0.997632 0.00417827 0.00417827 0.0105702 0.00417827 0.00417827 0.004039 0.0101417 EPI_ISL_412898_19_Wuhan_HBCDC-HB-02_2019 0.00514103 0.0116715 0.442901 0.0386272 0.0077713 0.00596808 0.43407 0.0109768 0.786061 0.0109768 0.00361262 0.736279 0.00458524 0.00707842 1.55997e-08 0.872725 0.87203 0.00652325 0.00499653 0.0115326 0.0112547 0.00596808 0.00652959 0.0106989 0.00652959 0.00596808 0.0070853 0.00721721 0.789276 0.997638 0.0116715 0.0116715 0.00527998 0.0116715 0.0116715 0.0115326 0.00514032 EPI_ISL_410721_19_pangolin_Guangdong_1_2019 0.736418 0.73887 0.736871 0.750556 0.736192 0.736017 0.752087 0.738453 0.83331 0.738592 0.736418 0.00375626 0.736418 0.7356 1.23633e-06 0.860261 0.858771 0.736017 0.736017 0.738731 0.73887 0.736017 0.736593 0.738314 0.736732 0.736017 0.737427 0.737266 0.833866 0.997774 0.738592 0.739009 0.736996 0.73887 0.739009 0.738731 0.736316 EPI_ISL_412899_19_Wuhan_HBCDC-HB-03_2019 0.00430735 0.0108378 0.442484 0.0394609 0.00707744 0.00513532 0.433931 0.0101431 0.786061 0.0101431 0.0044463 0.736279 0.00361262 0.00624566 1.42027e-08 0.872725 0.87203 0.00569049 0.00582929 0.0108378 0.010421 0.00513532 0.00569603 0.00986522 0.00569603 0.00513532 0.00611281 0.00638446 0.789415 0.997638 0.0108378 0.0108378 0.00611366 0.0108378 0.0108378 0.0106989 0.00430675 EPI_ISL_402121_19_Wuhan_IVDC-HB-05_2019 0.00652325 0.0130465 0.442887 0.0416378 0.00693866 0.00485774 0.435392 0.0123525 0.785229 0.0122137 0.00666204 0.7356 0.00582929 0.00360861 1.58325e-08 0.872866 0.872172 0.0055517 0.00569049 0.0129077 0.0124913 0.00499653 0.00721721 0.0120749 0.00707842 0.00485774 0.00721721 0.00610687 0.789035 0.997641 0.0130465 0.0129077 0.00832755 0.0127689 0.0129077 0.0127689 0.00569049 EPI_ISL_417385_19_Australia_NSW19_2020 1.53668e-08 2.23517e-08 7.42963e-07 7.1479e-08 1.74623e-08 1.46683e-08 7.28061e-07 2.11876e-08 1.31759e-06 2.11876e-08 1.58325e-08 1.23563e-06 1.44355e-08 1.6531e-08 -0.999998 1.46171e-06 1.46055e-06 1.55997e-08 1.58325e-08 2.18861e-08 2.16532e-08 1.46683e-08 1.69966e-08 2.16532e-08 1.72295e-08 1.46683e-08 1.72295e-08 1.6531e-08 1.32364e-06 1.66777e-06 2.21189e-08 2.21189e-08 1.72295e-08 2.21189e-08 2.18861e-08 2.18861e-08 1.44355e-08 EPI_ISL_410539_19_pangolin_Guangxi_P1E_2017 0.871474 0.872497 0.878161 0.877364 0.871635 0.871339 0.88223 0.872497 0.898711 0.872497 0.871335 0.863042 0.871335 0.871339 1.45962e-06 0.00375417 0.0145955 0.871478 0.871478 0.872497 0.872775 0.871478 0.871353 0.872219 0.87177 0.871339 0.871631 0.871617 0.898875 0.997497 0.872497 0.872775 0.871802 0.872636 0.872497 0.872775 0.871492 EPI_ISL_410540_19_pangolin_Guangxi_P5L_2017 0.870502 0.871699 0.876493 0.876564 0.870525 0.870645 0.880873 0.87156 0.899543 0.871699 0.870502 0.861134 0.870502 0.870507 1.45822e-06 0.0147345 0.00361412 0.870645 0.870507 0.871699 0.871977 0.870645 0.87052 0.871421 0.870936 0.870645 0.870659 0.870784 0.899014 0.997637 0.871699 0.871838 0.871004 0.871838 0.871699 0.871977 0.870659 EPI_ISL_403930_19_Wuhan_IPBCAMS-WH-03_2019 0.00610687 0.0124913 0.442609 0.0405274 0.00638357 0.00444136 0.434976 0.0117974 0.786061 0.0117974 0.00624566 0.736017 0.00541291 0.00569049 1.5134e-08 0.873282 0.872588 0.0037474 0.00527412 0.0123525 0.0120749 0.00458015 0.00680083 0.0115198 0.00666204 0.00444136 0.00680083 0.00569049 0.789729 0.997779 0.0124913 0.0123525 0.00791117 0.0123525 0.0123525 0.0122137 0.00527412 EPI_ISL_434534_19_Wuhan_IVDC-HB-GX02_2019 0.00624566 0.0127689 0.442609 0.0396947 0.00679989 0.00458015 0.434698 0.0120749 0.786061 0.0120749 0.00485774 0.735878 0.00569049 0.00582929 1.53668e-08 0.873005 0.872311 0.00527412 0.0037474 0.0124913 0.0123525 0.00471895 0.00707842 0.0117974 0.00693963 0.00458015 0.00707842 0.00596808 0.789452 0.997779 0.0127689 0.0127689 0.00652325 0.0126301 0.0126301 0.0124913 0.0055517 EPI_ISL_471189_19_USA_WI-WSLH-200024_2020 0.0111157 0.00487465 0.444985 0.0412256 0.0135998 0.0116586 0.435237 0.00431695 0.788416 0.00417827 0.0112547 0.738314 0.01056 0.0127689 2.14204e-08 0.873888 0.873228 0.0122137 0.0124913 0.00362117 0.00445682 0.0116586 0.0122256 0.00487329 0.0122256 0.0116586 0.0122256 0.0129077 0.791221 0.997493 0.00487465 0.00473538 0.0112656 0.00473538 0.00473538 0.0045961 0.0106974 EPI_ISL_422680_19_Netherlands_NA_136_2020 0.0108378 0.00473604 0.445263 0.0412371 0.013461 0.011381 0.435358 0.00431695 0.788555 0.00417827 0.0111157 0.738731 0.0102821 0.0126301 2.14204e-08 0.873888 0.873228 0.0119362 0.0122137 0.00473538 0.00362218 0.011381 0.0119478 0.00459482 0.0120867 0.011381 0.0120867 0.0126301 0.79136 0.997492 0.00487465 0.00487465 0.0109875 0.00473538 0.00473538 0.00473538 0.0105585 MN908947.3_alt._SARS_CoV_2 0.00541291 0.0119362 0.442193 0.0405274 0.00582848 0.00388619 0.434559 0.011381 0.786061 0.011381 0.0055517 0.735878 0.00485774 0.00499653 1.42027e-08 0.873144 0.872311 0.00444136 0.00458015 0.0117974 0.0116586 0.00388619 0.00624566 0.0109646 0.00610687 0.00388619 0.00624566 0.00513532 0.789591 0.997779 0.0119362 0.0119362 0.007356 0.0119362 0.0119362 0.0117974 0.00471895 EPI_ISL_402128_19_Wuhan_WIV05_2019 0.00597388 0.0125035 0.442484 0.0407058 0.00818762 0.00624566 0.434982 0.0116699 0.786199 0.0118088 0.00611281 0.736177

0.00527924 0.0074948 1.67638e-08 0.872881 0.872326 0.00680083 0.00707842 0.0122256 0.0120867 0.00624566 0.00361211 0.011531 0.00694637 0.00624566 0.00736316 0.00763359 0.789415 0.997638 0.0123645 0.0123645 0.00777994 0.0123645 0.0123645 0.0122256 0.00541817 EPI_ISL_421370_19_USA_NY-PV08402_2020 0.0100042 0.00459482 0.444846 0.0409357 0.0126284 0.0105482 0.435394 0.00403787 0.788001 0.00403787 0.0102821 0.738036 0.00944838 0.0117974 2.07219e-08 0.873471 0.872811 0.0111034 0.011381 0.00445558 0.00431635 0.0105482 0.0112531 0.00362016 0.0113921 0.0105482 0.0112531 0.0117974 0.790804 0.997633 0.00459482 0.00459482 0.0102921 0.00459482 0.00459482 0.00445558 0.00986385 EPI_ISL_402130_19_Wuhan_WIV07_2019 0.00597388 0.0125035 0.443179 0.0409836 0.00804885 0.00582929 0.434287 0.0116699 0.786892 0.0118088 0.00625174 0.736316 0.00527924 0.00721721 1.67638e-08 0.873159 0.872465 0.00666204 0.00680083 0.0123645 0.0120867 0.00582929 0.00680745 0.011531 0.00361211 0.00582929 0.00722423 0.007356 0.790249 0.997777 0.0125035 0.0125035 0.00777994 0.0125035 0.0123645 0.0123645 0.00527924 EPI_ISL_402119_19_Wuhan_IVDC-HB-01_2019 0.00527412 0.0119362 0.442193 0.0403886 0.00596725 0.0037474 0.434559 0.0112422 0.786061 0.0112422 0.0055517 0.735878 0.00471895 0.00499653 1.42027e-08 0.873005 0.872311 0.00444136 0.00458015 0.0117974 0.0115198 0.00388619 0.00624566 0.0109646 0.00610687 0.0037474 0.00610687 0.00499653 0.789452 0.997641 0.0119362 0.0119362 0.00721721 0.0117974 0.0117974 0.0116586 0.00458015 EPI_ISL_406798_19_Wuhan_WH01_2019 0.00652959 0.0123645 0.442206 0.04015 0.00832639 0.00610687 0.434982 0.0116699 0.786338 0.0116699 0.00666852 0.736732 0.00583495 0.00721721 1.6531e-08 0.872881 0.872187 0.00680083 0.00680083 0.0122256 0.0119478 0.00624566 0.00722423 0.0113921 0.0070853 0.00610687 0.00375104 0.007356 0.789693 0.997638 0.0123645 0.0122256 0.00764101 0.0122256 0.0122256 0.0120867 0.00569603 EPI_ISL_403931_19_Wuhan_IPBCAMS-WH-02_2019 0.00666204 0.0131853 0.443303 0.0403886 0.00721621 0.00513532 0.43567 0.0124913 0.786476 0.0124913 0.00693963 0.736849 0.00610687 0.00652325 1.62981e-08 0.873282 0.872588 0.00582929 0.00596808 0.0130465 0.0127689 0.00527412 0.0074948 0.0122137 0.007356 0.00527412 0.0074948 0.0037474 0.790007 0.997641 0.0131853 0.0130465 0.00860514 0.0130465 0.0130465 0.0130465 0.00582929 MG772934.1_bat_SL_CoVZXC21 0.790388 0.791916 0.793721 0.801639 0.790591 0.790423 0.793444 0.792054 0.308716 0.792054 0.790249 0.833588 0.790388 0.790007 1.32504e-06 0.898041 0.898319 0.790562 0.790423 0.792054 0.792332 0.790423 0.790249 0.791777 0.79136 0.790423 0.790804 0.790978 0.00361161 0.997639 0.792193 0.792471 0.791082 0.792332 0.792054 0.792471 0.790388 humanMito 0.998194 0.998328 0.999028 0.998011 0.998335 0.998334 0.998465 0.99819 0.998753 0.998189 0.998194 0.999026 0.998194 0.998196 1.66893e-06 0.998888 0.998888 0.998473 0.998334 0.998189 0.998189 0.998334 0.998194 0.998329 0.998194 0.998334 0.998472 0.998334 0.99875 0.00602107 0.998189 0.998189 0.99847 0.998329 0.998189 0.998189 0.998194 EPI_ISL_420376_19_Belgium_CF-0324119_2020 0.0112547 0.00501393 0.444846 0.041922 0.0137386 0.0117974 0.435515 0.0044562 0.788693 0.00431755 0.0115326 0.738314 0.0106989 0.0129077 2.16532e-08 0.874027 0.873228 0.0123525 0.0126301 0.00487465 0.0045961 0.0117974 0.0123645 0.00501253 0.0125035 0.0117974 0.0123645 0.0130465 0.79136 0.997493 0.00362117 0.00501393 0.0114047 0.00501393 0.00501393 0.00487465 0.0109753 EPI_ISL_437037_19_Denmark_ALAB-SSI-679_2020 0.0111157 0.00487465 0.444429 0.0416435 0.0135998 0.0116586 0.43468 0.00431695 0.788832 0.00417827 0.0112547 0.738731 0.010421 0.0127689 2.14204e-08 0.874027 0.873367 0.0122137 0.0123525 0.00473538 0.00445682 0.0116586 0.0122256 0.00487329 0.0122256 0.0116586 0.0122256 0.0129077 0.791499 0.997632 0.00487465 0.00362117 0.0111266 0.00473538 0.00473538 0.00473538 0.0106974 EPI_ISL_402127_19_Wuhan_WIV02_2019 0.00680839 0.0115438 0.443595 0.0386648 0.00929781 0.007356 0.434214 0.0105702 0.786476 0.0108484 0.00541892 0.736718 0.00625261 0.00846634 1.67638e-08 0.872914 0.872255 0.00791117 0.00638446 0.0114047 0.0111266 0.007356 0.00791887 0.0105702 0.00805779 0.007356 0.00805779 0.00860514 0.789971 0.997636 0.0114047 0.0114047 0.00361613 0.0114047 0.0114047 0.0112656 0.00652959 EPI_ISL_467889_19_USA_NY-QDX-136_2020 0.0111157 0.00487465 0.445402 0.0417827 0.0135998 0.0116586 0.435655 0.00431695 0.788555 0.00417827 0.0112547 0.738592 0.010421 0.0127689 2.14204e-08 0.873888 0.873228 0.0122137 0.0123525 0.00473538 0.00445682 0.0116586 0.0122256 0.00473406 0.0122256 0.0116586 0.0122256 0.0129077 0.79136 0.997632 0.00473538 0.00473538 0.0112656 0.00362117 0.00473538 0.0045961 0.0106974 EPI_ISL_484714_19_USA_MI-UM-2006S000857_2020 0.0111157 0.00487465 0.444985 0.0417827 0.0135998 0.0116586 0.435515 0.00431695 0.788416 0.00417827 0.0112547 0.738592 0.01056 0.0127689 2.14204e-08 0.873888 0.873089 0.0122137 0.0124913 0.00473538 0.00445682 0.0116586 0.0122256 0.00473406 0.0122256 0.0116586 0.0122256 0.0127689 0.791082 0.997493 0.00473538 0.00473538 0.0112656 0.00473538 0.00362117 0.0045961 0.0106974 EPI_ISL_487453_19_Mali_M002667_2020 0.0109768 0.00473538 0.444568 0.0416435 0.013461 0.0115198 0.435515 0.00417769 0.788416 0.004039 0.0111157 0.738314 0.0102821 0.0126301 2.11876e-08 0.874027 0.873367 0.0120749 0.0123525 0.0045961 0.00431755 0.0115198 0.0119478 0.00473406 0.0120867 0.0115198 0.0120867 0.0127689 0.791499 0.997493 0.00473538 0.00473538 0.0111266 0.0045961 0.0045961 0.00348189 0.0105585 EPI_ISL_402129_19_Wuhan_WIV06_2019 0.00444568 0.0109753 0.442206 0.0395943 0.00666112 0.00471895 0.433871 0.0102806 0.786061 0.0102806 0.00472353 0.736038 0.00388997 0.00596808 1.42027e-08 0.872742 0.872048 0.00527412 0.00541291 0.0109753 0.0105585 0.00471895 0.00527924 0.0100028 0.00514032 0.00471895 0.00583495 0.00596808 0.789415 0.997499 0.0109753 0.0109753 0.00625174 0.0109753 0.0109753 0.0108363 0.00361211

V. The Figures in the Main Paper Text

The Figures in the main paper text can not be expanded in hard copy. But the Supplementary Information (SI) is an on-line Appendix and can be enlarged. Hence we supply these Figures here.

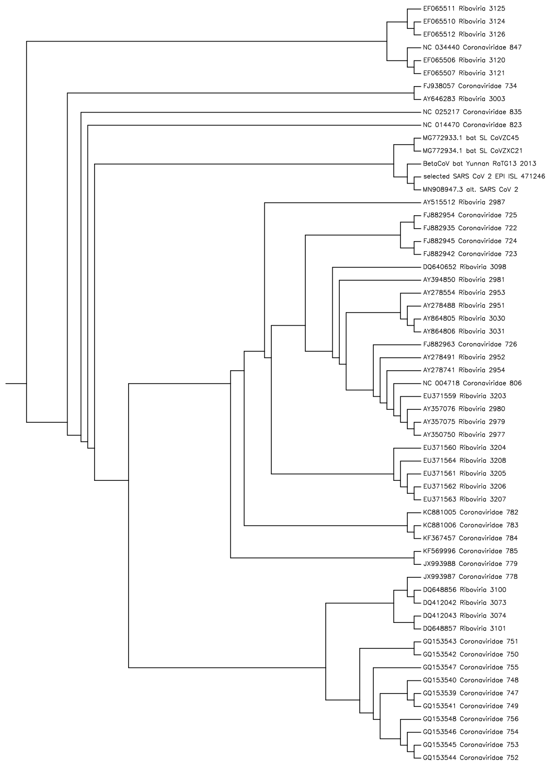

Figure S3. Figure 1 in the main text of the paper

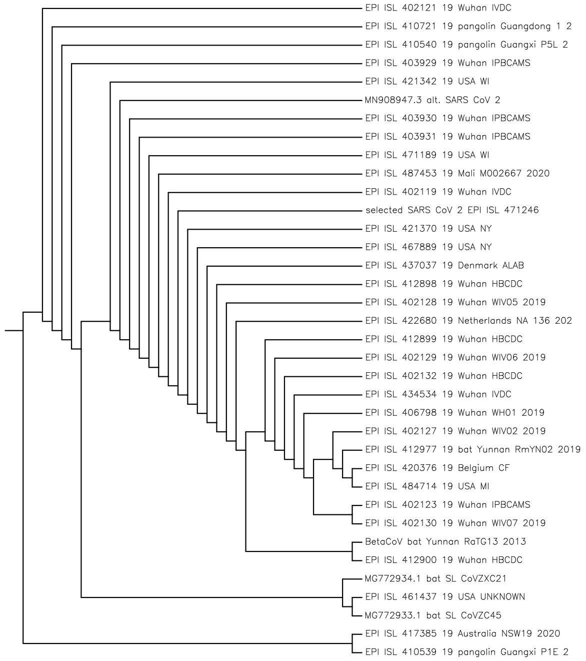

Figure S4. Figure 2 in the main text of the paper
